# Keynotes to the Materia Medica

**Published:** 1887-04

**Authors:** 


KEYNOTES TO THE MATERIA MEDICA, as taught by Henry N. Guernsey, M. D. Edited by Joseph C. Guernsey, A. M., M. D. Philadelphia: F. E. Bœricke, Hahnemann Publishing House, 1887.

The book before us purports to be a *resumé* of Dr. Guernsey's lectures on materia medica in Hahnemann College. Such it is. But it is especially devoted to his famous "keynotes," so valuable to every practitioner, and first published in his well-known work on obstetrics. As was said by Dr. Nash in a recent letter to the editor of this journal, "No man is fit to practice medicine until he knows these keynotes by heart."

Well, here they are, collected by the industry of Dr. Guernsey's son, who, by the way, reported the lectures at the time of their delivery. In these lectures the younger Dr. Guernsey had a grand opportunity for a perfect repertory to the keynotes exclusively. We regret to say he has not embraced it. The repertory appended is written in the same old ruts, much of it copied from Bœnninghausen, and arranged by regions, as in the *Materia Medica*, instead of being rigidly alphabetical.

